# Isolated rupture of the gallbladder following blunt abdominal trauma: case report

**DOI:** 10.1590/S1679-45082013000200016

**Published:** 2013

**Authors:** Marina Gabrielle Epstein, Dorivaldo Lopes da Silva, Naim Carlos Elias, Gustavo Tricta Augusto Sica, Murillo de Lima Fávaro, Marcelo Augusto Fontenelle Ribeiro

**Affiliations:** 1 Universidade de Santo Amaro, São Paulo, SP, Brazil; 2 Hospital Geral do Grajaú, São Paulo, SP, Brazil; 3 Universidade de Santo Amaro, São Paulo, SP, Brazil; Hospital Geral Dr. Moysés Deutsch, São Paulo, SP, Brazil.; 4 Hospital Geral Dr. Moysés Deutsch, São Paulo, SP, Brazil

**Keywords:** Gallbladder/injuries, Abdominal injuries, Laparotomy/methods, Cholecystectomy, Case reports

## Abstract

Gallbladder rupture following blunt abdominal trauma is a rare event recognized on evaluation and treatment of other visceral injuries during laparotomy. Isolated gallbladder rupture secondary to blunt abdominal trauma is even more uncommon. The clinical presentation of gallbladder injury is variable, resulting in a delay in diagnosis and treatment. We report the case of a patient who suffered an isolated gallbladder rupture due to blunt abdominal trauma.

## RESUMO

A ruptura da vesícula biliar após trauma abdominal fechado é um evento raro, identificada apenas durante laparotomia para avaliar e tratar outras lesões viscerais. A ruptura isolada da vesícula biliar secundária a traumatismo abdominal fechado é ainda mais rara. A apresentação clínica da lesão da vesícula biliar é variável, resultando em um atraso no diagnóstico e tratamento. Relata-se um caso de ruptura isolada da vesícula biliar devido a um trauma abdominal fechado.

## INTRODUCTION

Isolated traumatic gallbladder injuries are uncommon and difficult to diagnose^(1)^. Gallbladder injury arises from compressive and shearing forces, most commonly in motor vehicle accidents. Computed tomography is the best technique for diagnosing gallbladder injury. The treatment of choice is cholecystectomy^(2)^.

## CASE REPORT

AGF, a 27-year-old male, presented at the Emergency Room of *Hospital Geral do Grajaú* complaining of abdominal pain for 3 weeks, which had worsened over the past 2 days. He reported no fever, vomiting or changes in bowel movements. He reported that 28 days prior he had fallen from a motorcycle and had been seen at another service where an abdominal computed tomography scan was performed due to abdominal pain. It revealed a small amount of free fluid. He remained hemodynamically stable and was discharged.

On examination, he was in good general health conditions, afebrile, icteric and hemodynamically stable. The abdomen was tender on diffuse palpation and rebound tenderness.

**Figure 1:**
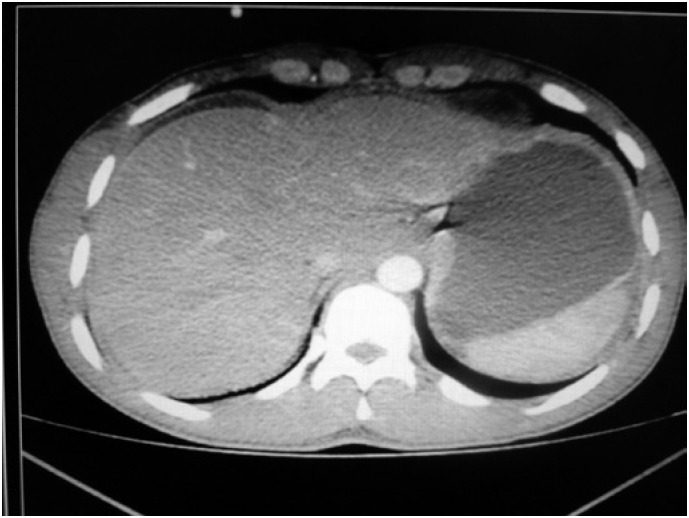
Computed tomography showed a large amount of free fluid

The laboratory test results were hemoglobin 14.2mg/dL, leukocytes 8,200 with no shifts, AST 65U/L, ALT 157U/L, alkaline phosphatase 371U/L, gamma GT 384U/L, total bilirubin 4.2mg/dL and amylase 104 U/L. Another abdominal CT was ordered and showed a large amount of free fluid (Figure 1).

**Figure 2:**
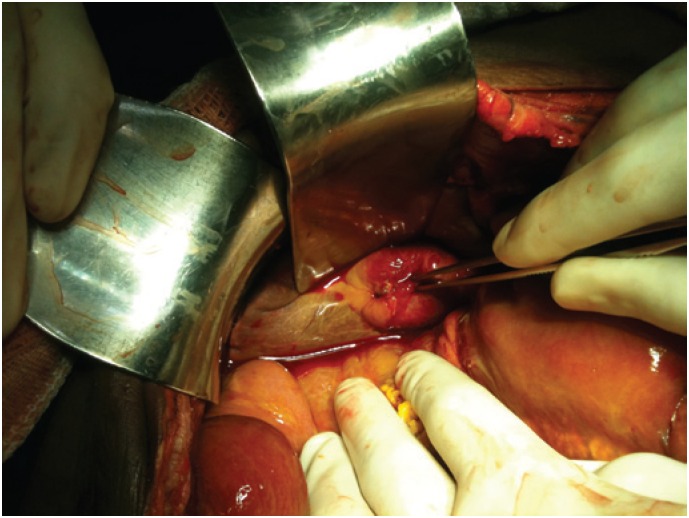
Isolated perforation of the gallbladder

Exploratory laparotomy was recommended, which identified extensive bile peritonitis and an isolated perforation in the bottom of the gallbladder (Figure 2). Other hollow and parenchymatous organs showed no sign of injury.

Cholecystectomy was performed, followed by draining of the cavity.

The patient recovered uneventfully and was discharged on the third postoperative day, with antibiotic therapy. At the time we reported this case, the patient was on postoperative day 20, asymptomatic, and followed on an outpatient basis.

## DISCUSSION

Injuries in the gallbladder resulting from blunt abdominal trauma occur rarely and are usually associated with damage to other abdominal organs^(1)^. Isolated perforation of the gallbladder is uncommon due to its protected anatomical position.

Traumatic injury of the gallbladder is unusual. Its overall incidence varies from 0.8 to 2.1% in patients who suffered some type of abdominal trauma; 2% of patients undergoing laparotomy for traumatic injury are found to have a gallbladder injury^(3)^.

Most gallbladder injuries occur following motor vehicle accidents, significant falls, and direct blows in sports.

Gallbladder perforation is more likely in cases when the gallbladder is distended and thin-walled at the time of injury. Although there has been an isolated case of injury secondary to a bull head-butting a patient's abdomen, there are no identifiable cases of damage occurring due to this mechanism^(3)^.

The presence of sterile bile in the peritoneal cavity causes a slight irritation, which explains the slow evolution. The patient may present with jaundice, which is due to the absorption of bile pigments by the peritoneum, as in this case^(4)^.

The diagnosis of the gallbladder lesion resulting from blunt abdominal trauma is usually confirmed during exploratory laparotomy. Frequently other more severe lesions in organs overlapping the gallbladder are observed^(5)^.

When laparotomy is not done, there may be an interval of 1 to 6 weeks to diagnose a traumatic injury of the gallbladder, like in this case.

Cholecystectomy is the recommended treatment for gallbladder rupture and major tearing. Partial cholecystectomy has also been described in the literature and is an option in selected cases. Laparoscopic cholecystectomy is advocated as a safe and effective procedure for diagnosis and management of traumatic gallbladder rupture^(6)^. In this case, due to uncertain diagnosis, an exploratory laparotomy was chosen as the safest option.

## References

[1] Liess BD, Awad ZT, Eubanks WS (2006). Laparoscopic cholecystectomy for isolated traumatic rupture of the gallbladder following blunt abdominal injury. J Laparoendosc Adv Surg Tech A.

[2] Salomao RM, Magalhães NC, Silva FV, Iglesias AC (2008). Colecistite aguda decorrente de hemorragia intraluminar da vesícula biliar após trauma abdominal fechado. Rev Col Bras Cir.

[3] Søndenaa K, Horn A, Nedrebø T (2000). Diagnosis of blunt trauma to the gallbladder and bile ducts. Eur J Surg.

[4] Zantut LFC, Machado MAC, Volpe P, Poggetti RS, Birolini D (1995). Lesões traumáticas da vesícula e trato biliar extra-hepático: análise de 45 casos. Rev Assoc Med Bras.

[5] De Raet J, Lamote J, Delvaux G (2010). Isolated traumatic gallbladder rupture. Acta Chir Belg.

[6] Bainbridge J, Shaaban H, Kenefick N, Armstrong CP (2007). Delayed presentation of an isolated gallbladder rupture following blunt abdominal trauma: a case report. J Med Case Rep.

